# The Influence of Typhoon Khanun on the Return Migration of *Nilaparvata lugens* (Stål) in Eastern China

**DOI:** 10.1371/journal.pone.0057277

**Published:** 2013-02-21

**Authors:** Gao Hu, Fang Lu, Ming-Hong Lu, Wan-Cai Liu, Wei-Gen Xu, Xue-Hui Jiang, Bao-Ping Zhai

**Affiliations:** 1 Key Laboratory of Integrated Management of Crop Diseases and Insect Pests (Ministry of Education), College of Plant Protection, Nanjing Agricultural University, Nanjing, China; 2 Division of Pest Forecasting, China National Agro-Tec Extension and Service Center, Beijing, China; 3 Plant Protection Station of Zhejiang Province, Hangzhou, China; Swedish University of Agricultural Sciences, Sweden

## Abstract

Migratory insects adapt to and exploit the atmospheric environment to complete their migration and maintain their population. However, little is known about the mechanism of insect migration under the influence of extreme weather conditions such as typhoons. A case study was conducted to investigate the effect of typhoon Khanun, which made landfall in the eastern China in Sept. 2005, on the migration of brown planthopper, *Nilaparvata lugens* (Stål). The migration pathways of *N. lugens* were reconstructed for the period under the influence of the typhoon by calculating trajectories using the MM5, a mesoscale numerical weather prediction model, and migration events were examined in 7 counties of the Yangtze River Delta region with ancillary information. The light trap catches and field observations indicated that the migration peak of *N. lugen*s coincided with the period when the typhoon made landfall in this region. The trajectory analyses revealed that most emigrations from this region during this period were hampered or ended in short distances. The sources of the light-trap catches were mainly located the nearby regions of each station (i.e. mostly less than 100 km away, with a few exceeding 200 km but all less than 300 km). This disrupted emigration was very different from the usual *N. lugens* migration which would bring them to Hunan, Jiangxi, and southern Anhui from this region at this time of year. This study revealed that the return migration of *N. lugens* was suppressed by the typhoon Khanun, leading to populations remaining high in the Yangtze River Delta and exacerbating later outbreaks.

## Introduction

Many insects undertake regular long-distance migrations each year to track seasonal changes in resources and habitats. By spreading their breeding efforts in space and time, migratory insects maintain their populations over a range of environmental conditions [1]. Various features of the atmospheric environment affect insect migration at different spatial scales. In most cases, migratory insects adapt to these features to maximise their flight efficiency and succeed in spatial redistribution [2]–[4]. Hence, prevailing seasonal airflows are crucial for annual regular migrations [4]–[7]. As aerial densities of migrating insects may be increased by strong wind convergence, migrants may land in favourable or unfavourable places at large number, either building up rapidly or resulting in high mortality. Therefore, migration is essentially a high-risk strategy [8], [9], especially under extreme weather, such as typhoons. Under the influence of such a weather system, usual migration routes are often perturbed and migrants may not reach a suitable habitat. Although there have been several studies of rice planthopper migration under the influence of typhoons (see below), little is known about changes in insect migration process and much less about the changes in density and distribution of migratory population under extreme weathers. Therefore, evaluation of the influence of extreme weather on insect migration is of great significance.

Brown planthopper, *Nilaparvata lugens* (Stål) (Hemiptera: Delphacidae), one of the most serious rice pests in East and Southeast Asia, undertakes an annual return migration in East Asia [10]–[12]. *N. lugens* cannot overwinter in temperate zones such as mainland China, Japan, and the Korean Peninsula. Instead, an infestation is initiated by windborne spring/summer migrants from the south [13], [14]. It begins with a northward migration occurring in late March every year. The distribution of *N. lugens* then further expands northward by progeny of the migrant population and can cover the whole rice-growing region in China, as well as into Japan and the Korean Peninsula. From September onwards the general direction of planthopper migration becomes predominantly south-bound. In the end of October, most *N. lugens* populations are only present in the safe overwintering areas, mainly in the Indo-China Peninsula, within which there is a proportion of successfully returned migrants [10], [13], [15], [16]. In the past few years new outbreaks of *N. lugens* have been continuously recorded. For example, the loss of rice yield in China caused by *N. lugens* was approximately 1,880,000 t in 2005 [16]–[18].

*N. lugens* is a micro insect and a slow flyer of only 0.3 m/s [19] and its windborne migration is significantly influenced by meteorological factors and phenomena. Take-off usually occurs in a narrow time window at dusk or dawn under calm weather, temperatures greater than 17°C, and wind speeds less than 3.1 m/s [20]–[23]. Long-distance displacement is induced by a long-lasting warm and humid wind. A low-level jet sustained for a long time over a wide scale is the most favourable current for northward migrations [15], [22], [24]–[28]. Landing is often observed under windy, rainy, or even stormy weather when a frontal system passes over regardless of the time of the day. Downdrafts also can terminate a migration, especially during southward migration [22], [24], [25], [29]–[31].

A typhoon, which can produce substantial winds of 250 kilometres per hour (kph) with gusts of up to 300 kph, as well as intense bursts of rain and ocean surges that cause extensive flooding, may change the *N. lugens* population distribution patterns. A typhoon can last for weeks and cover thousands of miles. Therefore, it could reroute *N. lugens* migrations, and thus cause either sudden infestation or population collapse. For example, *N. lugens* could have unusually migrated from Philippines to Taiwan and southern China, assisted by the circulation of a typhoon [32], [33]. In China, about seven typhoons in average make landfall every year. Most typhoon landings occur from July to September, which partially overlaps with the period of *N. lugens* migration (April to October). The influence of typhoon on the migration of *N. lugens* has been studied since the 1970s. In 1976, the synchronous immigration of *N. lugens* in Zhejiang, Jiangsu, and Anhui provinces from Aug. 12 to 24 was thought to be correlated with the inverted trough produced by the thirteenth typhoon [34]. Wang et al. (1982) categorised typhoons into three types according to the location of typhoon landing, namely, Beibu Gulf, South China Sea, and East China Sea. Among these, the East China Sea type was thought to be favourable for the southward or southeastward migration of planthoppers from Jiang-Huai Valley (Central and North Jiangsu, Central and North Anhui) [35]. From 1991 to 2005, 52 typhoons had made landfall in China, 16 of which influenced *N. lugens* migrations [36]. The serious *N. lugens* outbreaks in 2005 have been attributed to the increased number and intensity of typhoons that made landfall in the year [18], [37], [38]. Nevertheless, knowledge of the influence of typhoon on planthopper migration remains limited and ambiguous, largely due to the complexity of typhoon's structure and the uncertainty of typhoon's movement.

This paper reports a case study on the effect of typhoon Khanun on the return migration of *N. lugens* in eastern China in 2005, in order to improve the understanding of the influence of typhoon on this pest's population distribution. In 2005, the population of *N. lugens* in the Yangtze River Valley increased exponentially in September. ‘Hopperburn’, which is browning of the leaves or withering of the whole plant, occurred widely in mid-September in the Yangtze River Delta (including Zhejiang and Jiangsu provinces and Shanghai municipality) [18], [37]–[39]. Based on previous researches, *N. lugens* in this region would undertake return migration in this time of year, and the local population would decrease and the usual destination would be Hunan, Jiangxi and southern Anhui provinces [13], [16], [23], [40]. Six typhoons made landfall between Mid July and Mid October 2005 in eastern China. Among of these, the intensity of typhoon Khanun was the strongest. It landed in Zhejiang in mid September when *N. lugens* population level was very high in the Yangtze River Delta [18], [37], [38], [41]. By investigating the influence of typhoon Khanun on the population dynamics and distribution of *N. lugens* in this region, this study aims to improve the understanding of the relationship between insect migration process and the meteorological background.

## Materials and Methods

### Light trap data and field observation data

**Figure 1:**
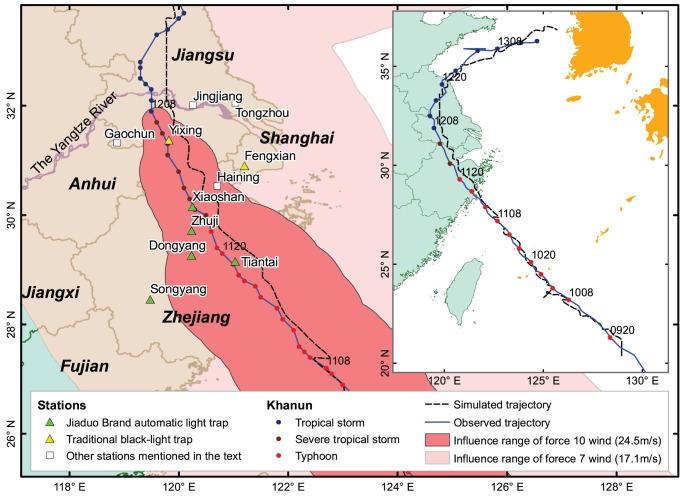
The trajectory and influence range of typhoon Khanun. Note: The numbers near track points represent time formatted as Day-House DDTT; for example, 0708 was at 0800 h on Sept. 7.

Daily light trap data from plant protection stations (PPS) for seven counties in the Yangtze Delta region, including Tiantai, Xiaoshan, Dongyang, Zhuji, Songyang, Fengxian and Yixing, were obtained from Zhejiang province, Shanghai municipality and Jiangsu province in 2005 (Fig. 1). In all but the Fengxian and Yixing PPS, Jiaduo Brand automatic light traps (black-light lamp with the power of 20 W, Jiaduo Brand, Jiaduo Science, Industry and Trade Co. Ltd., Henan Province, China) were used for catching planthoppers. Traditional black-light traps were used in Fengxian and Yixing. These lamps were switched on at 1900 h in the evening and off at 0700 h Beijing Time (same thereafter) in the next morning every day from Apr. 1 to Nov. 15. At the time when this study was done, there had been no comparative study on the efficacy of these two types of light trap for capturing *N. lugens*. Comparisons between light traps at different stations were not made in this study.

Field observations were also collected from these stations as well as other counties in this region. Most field observation data were obtained from China National Agro-Tec Extension and Service Center (NATESC), while others were collected from pest reports published by related PPS and research papers [37]–[39], [42].

### Meteorological data and model

MM5, which is a limited-area, nonhydrostatic, terrain-following sigma-coordinate model designed to simulate or predict mesoscale atmospheric circulation, was used in this study. MM5 has several advanced features, including multiple nesting capability, various global model output data input, physical parameterisation choices, and the capability to run on various computer platforms.

The MM5 model system includes TERRAIN, REGRID, LITTLE_R, INTERPF, MM5, INTERPB, and other programs. NCEP Final Analysis (FNL) Data, which is a six-hourly, global, 1-degree grid dataset, was used in REGRID to generate first-guess pressure-level fields on model grids. Routine sounding (six-hourly) and surface (three-hourly) observational data from China Meteorological Data Sharing Service System were used in LITTLE_R to perform objective analysis to improve the first-guess fields, and in MM5 to perform four-dimensional data assimilation (FDDA) to improve the simulation result.

The dimension and resolution of the domain are 75×75 and 30 km, respectively. Thirty-three vertical layers were available and the model top was 100 hPa. The scheme selection and parameters for MM5 are listed in Table 1. The forecast time is 72 h. The model outputs data at 1 h intervals, including horizontal wind speed, vertical wind speed, temperature, precipitation, and other elements. Output data from MM5 were transformed to GrADS grid data by MM5toGrADS to display in the Grid Analysis and Display System (GrADS).

**Table 1:** Selection of scheme and parameters for MM5.

Items	Domain1
Location	30°N, 119°E
The number of grid points	75×75
Distance (km) between grid points	30
Grid start point of nest relative to its mother	1, 1
Layers	33
Map projection	Lambert
3D FDDA (Four-Dimensional Data Assimilation)	True
SFC FDDA	True
Explicit moisture schemes	Mix phase
Nesting type	One-way nest
Cumulus parameterization	Grell
Planetary boundary layer scheme	MRF PBL
Radiation scheme	RRTM
Model Start Time	0800 h on Sept. 8 and 11 (BJT)
Forecast Time	72 h

Other observation weather data was collected from published research papers [41], [43], [44].

### Trajectory analysis

Likely sources and landing area of migrating *N. lugens* were estimated by constructing backward/forward trajectories, based on the following assumptions: (i) the planthoppers are displaced downwind [20]–[23], [40], [45]; (ii) take-off takes place mostly at dusk and partly at dawn [20]–[23], [40], [45] ; (iii) the migrants are likely to land at any time; and (iv) the planthoppers cannot fly in an atmosphere of below 16.5°C [28], [40], [46]. The backward trajectories from light trap locations were calculated hourly during 0700 h to 0600 h, with initial 7 heights of 500, 750, 1000, 1250, 1500, 1750, and 2000 m above mean sea level. Thus, 168 trajectories (24×7) would be calculated for each location in one day. These backward trajectories were terminated when the air temperature at a point falling below 16.5C or at the take-off time, which was about 1800 h. The dawn take-off was not considered as its number is much lower than that at dusk [20]–[23]. The trajectories were calculated up to 36 h [13]. Hence, the flight duration is (i) below 13 h if migrants take off at dusk of the same night; (ii) 13–24 h if migrants take off at the previous dusk and lands at daytime; and (iii) above 24 h if migrants take off at the previous dusk and lands at night. A trajectory endpoint is considered to be a likely source if it is located in a place that satisfies the following conditions: (i) under fine weather at dusk (the air temperature at 2 m above ground greater than 17°C, the hourly precipitation less than 0.1 mm and the wind speed at 10 m above ground less than 4 m/s, based on the output data from MM5) [20]–[23]; (ii) in a rice planting area where the crops are at later growth stage [13]. The calculation setup of forward trajectory was as same as that of backward trajectory except the initial time at dusk (about 1800 h).

Backward and forward trajectories were calculated based on the method introduced by Zhu and Liao (1992) with wind field and temperature data that were transformed from the MM5 output by MM5toGrADS [47]. In this method, the displacement of particle at (*i*, *j*, *k*) is calculated using the following equations:
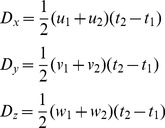
(1)where *D_x_*, *D_y_*, and *D_z_* denote the displacements in the *x*, *y*, and *z* dimensions, respectively, and (*u*_1_, *v*_1_, *w*_1_) and (*u*_2_, *v*_2_, *w*_2_) are wind velocities outputted by MM5 at times *t*_1_ and *t*_2_, respectively.

First, let (*u*_2_, *v*_2_, *w*_2_) be equal to (*u*_1_, *v*_1_, *w*_1_). The position of particles at *t*_2_ (*i*+*μ_x_*, *j*+*μ_y_*, *k*+*μ_z_*) was calculated using equation (1). The wind velocity (*u_i+μx_*,*v_j+μy_*, *w_k+μz_*) at position (*i*+*μ_x_*, *j*+*μ_y_*, *k*+*μ_z_*) at time *t*_2_ was calculated using the Lagrange interpolation formula. (*u*_2_, *v*_2_, *w*_2_) were then replaced by (*u_i+μx_*,*v_j+μy_*, *w_k+μz_*), that is, (*u_i+μx_*,*v_j+μy_*, *w_k+μz_*) is the zero-order approximation for (*u*_2_, *v*_2_, *w*_2_). Using equation (1) and the Lagrange interpolation formula iteratively, a series of approximations for (*u*_2_, *v*_2_, *w*_2_) could be calculated until the requirements listed by equation (2) were achieved.
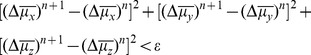
(2)where 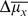, 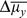 and 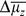 denote nondimensional displacements and are equal to the displacement divided by the grid distance in the *x*, *y*, and *z* dimensions, respectively. *n* and *n+*1 denote the *n* and *n*+1 order approximations, respectively. *ε*, the accuracy, was equal to 10^−6^ in this study.

The program for calculating trajectory was written in FORTRAN and run under Fedora 13 (Fedora Project, http://fedoraproject.org/).

### Ethics statement

No specific permits were required for the described field studies. The brown planthopper *Nilaparvata lugens* (Stål) is a major pest of rice in Asia, and lots of manpower and resources were used to control this pest every year.

In this study, it was confirmed that (i) the location is not privately-owned or protected in any way, and (ii) the field studies did not involve endangered or protected species.

## Results

The influence on the *N. lugens* migration from the typhoon Khanun had been examined according to the spatial relation of the study area and the typhoon. The possible sources of the immigrant *N. lugens* population were investigated with the MM5 model and ancillary information.

### Outline of typhoon Khanun and *N. lugens* occurrence in East China

Typhoon Khanun, the fifteenth to hit China in 2005, was formed in the eastern ocean of the Philippine Islands on Sept. 7 and made landfall the southeastern Zhejiang at 1451 h on Sept. 11. The Khanun quickly weakened as it moved over land. It went back to the sea again in the northern Jiangsu at 2000 h on Sept. 12 and then disappeared in the eastern Yellow Sea on Sept. 13. Zhejiang, Shanghai, Jiangsu, Anhui, and Shandong suffered strong winds, high waves, and heavy rains as the Khanun passed over those regions (Fig. 1).

**Figure 2:**
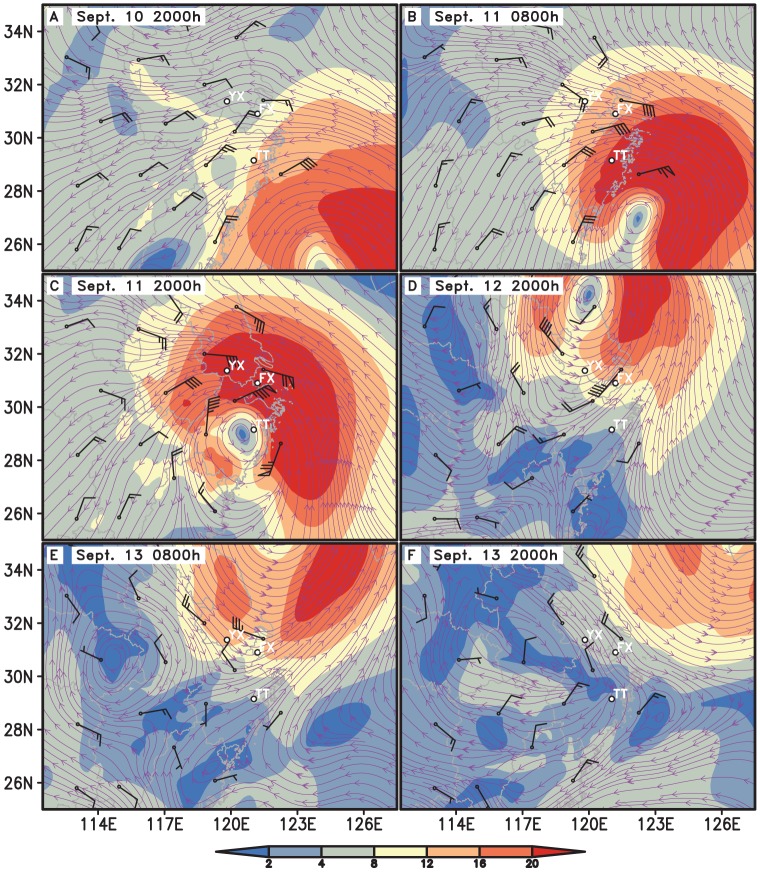
The simulated streamline and isotach of horizontal wind field (shaded), and the observed winds (shafts & barbs, whole barbs indicated 4 m/s) at 850 hPa. Note: TT-Tiantai, FX-Fengxian and YX-Yixing.

**Figure 3:**
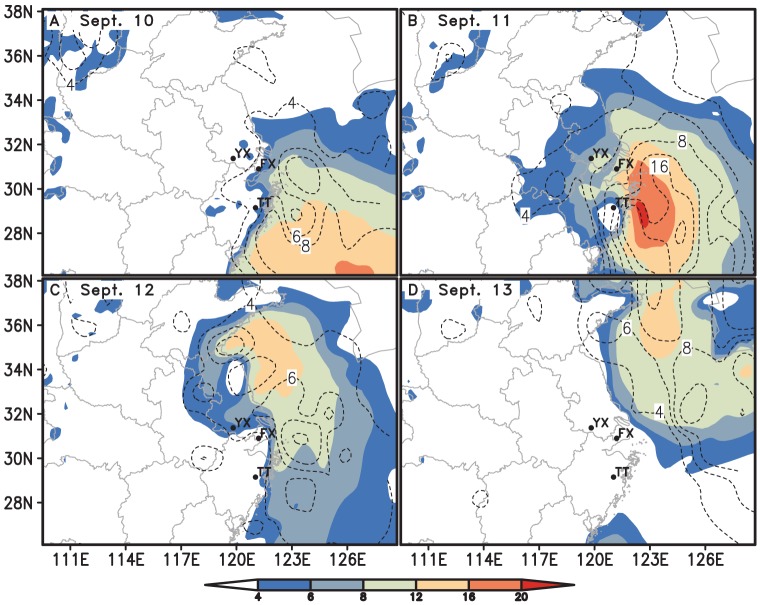
The simulated horizontal wind speeds at 10 m above ground at 1800 h (shaded, m/s) and the observed surface wind speeds at 1700 h (dotted line, m/s). Note: TT-Tiantai, FX-Fengxian and YX-Yixing.

The simulated trajectory of the typhoon by MM5 was similar to the observed. The error between the simulated and the observed trajectory on land was greater than that on the sea as land topography is more complicated. At the time when routine surface observation data exited, like at 0200, 0500, 0800 h, the point of simulated trajectory was much closer to that of the observed due to the improvement made by LITTLE_R and FDDA modules. (Fig. 1). Compared with the routine sounding data, the simulated wind speeds and directions agreed generally with the observed values (Fig. 2). At the take-off time of *N. lugens*, 1800 h, there was no observational meteorological data to compare. However, the simulated winds near surface at 1800 h were seemingly credible as they were close to the observed surface wind at 1700 h (Fig. 3).

**Figure 4:**
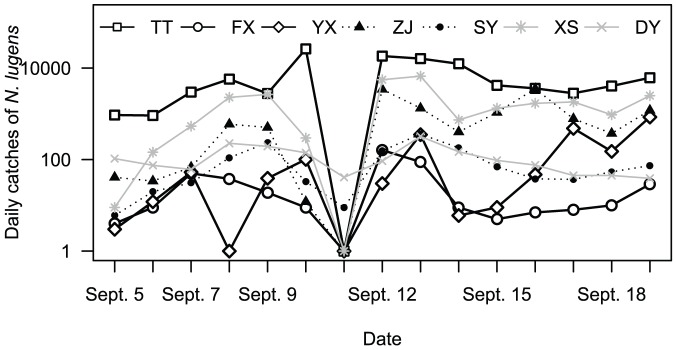
The daily light trap catches of *N. lugens* in Tiantai (TT), Fengxian (FX), Yixing (YX), Zhuji (ZJ), Songyang (SY), Xiaoshan (XS) and Dongyang (DY) in mid Sept., 2005.

The Khanun landing coincided with the peak of the (migratory) macropterous adults of *N. lugens* in the Yangtze Delta region. From the field observations, the percentage of macropters in total adults was increasing up to ∼70% in early September (Table 2). The captures of *N. lugens* by the light traps in this region also indicated this trend (Fig. 4), though it is hardly to tell if they were from local population or immigrants (see later analysis). The deep valleys of light-trap capture occurred on Sept. 10 (to 12) were simply due to the impact on the light-trap efficiency from the strong winds and torrential rains of the Khanun. However, the number of *N. lugens* adults in field increased in most counties in this region right after the Khanun passed (Table 2). For example, in Zhejiang and Jiangsu, there were five out of seven counties where the number of *N. lugens* in ‘systematic observation’ paddies increased during the typhoon period. Among these five counties except Jingjiang, the number increased by nearly 2 folds and higher. In the other two counties, the total numbers of *N. lugens* in ‘systematic observation’ paddies decreased, but the numbers of adults increased markedly. Likewise, there were six out of eight counties where the total number of *N. lugens* in farmer's paddies increased. However, the increase of *N. lugens* was small in Tiantai, Xiaoshan and Tongzhou, and was obvious in Zhuji, Yixing and Jingjiang. In Shanghai, the numbers of *N. lugens* in both ‘systematic observation’ paddies and farmer's paddies were decreased (Table 2). Above all, the local population of *N. lugens* seemed increased by the Khanun.

**Table 2:** Population density of *N. lugens* before & after the typhoon Khanun in 2005.

Province	County	Paddy type*	The number of *N. lugens* per 100 hills					Increase or not
			Before Khanun			After Khanun		
			Aug. 30	Sept. 5	Sept. 10	Sept. 15	Sept. 20	
Zhejiang	Xiaoshan	S	235	1055	1985	16420	22975	↑
	Haining	S	314	163	-	1603	1986	↑
		AS(M)	16(13)	34(12)	-	102(19)	130(66)	↑
	Dongyang	S	174	811	2004	1168	578	↓
		AS(M)§	257(252)	32(22)	20(16)	1300(1260)	488(420)	↑
Jiangsu	Yixing	S	650	10660	13500	7440	-	↓
		AS(M)	-	510(70)	75(70)	210(190)	-	↑
	Tongzhou	S	2522	1284	1730	5000	2445	↑
	Gaochun	S	1740	2660	3900	7080	18260	↑
	Jingjiang	S	507	475	10930	12713	21888	↑
Shanghai	Fengxian	S	622	9439	11372	6431	9993	↓
Zhejiang	Tiantai	F	-	2856	3877	4644	-	↑
		AF(M)	-	499(441)	286(209)	364(291)	-	↑
	Zhuji	F	1710	1333	5902	12422	12372	↑
		AF(M)	446(223)	602(429)	812(654)	857(712)	1159(1063)	↑
	Xiaoshan	F	25	23	100	390	367	↑
		AF(M)	23(23)	17(17)	22(22)	57(57)	67(63)	↑
	Dongyang	F	670	2250	3700	2210	1130	↓
Jiangsu	Yixing	F	28.9	1232	1918	7130	-	↑
	Tongzhou	F	376	517	858	894	2527	↑
	Gaochun	F§	1211	2315	1410	997	678	↓
	Jingjiang	F§	116	-	473	1511	4651	↑
Shanghai	Fengxian	F	467	2766	5030	3606	3575	↓

* S-Systematic investigation paddies which are moderately fertile with routine cultural practices and no pesticide use to control pests during the rice growing season; F-Famers' paddies; AS-Adults in systematic investigation paddies; AF-Adults in farmer's paddies; M-Macropterous adult.

§ The number of rice planthoppers, including *N. lugens* and *Sogatella furcifera* (Horváth), however, *N. lugens* account for more 80%.

### Before the approach of the Khanun

**Figure 5:**
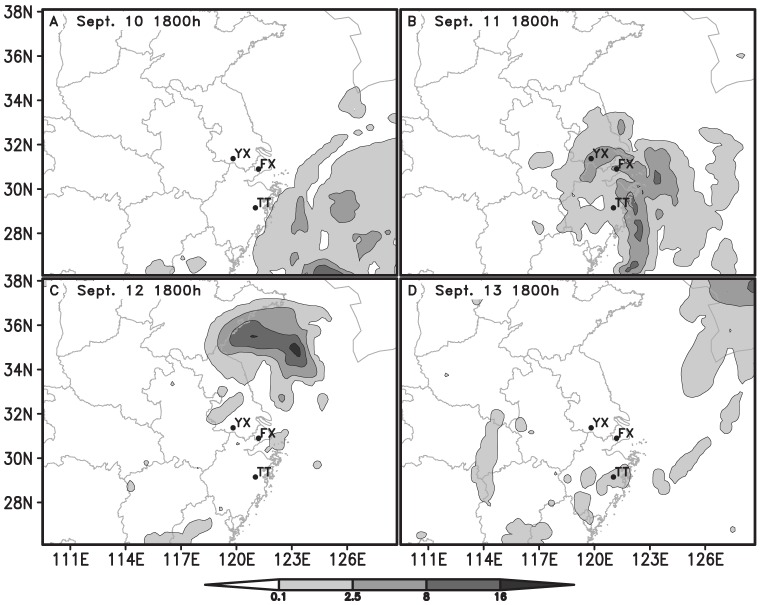
The simulated precipitation (mm) at 1800 h. Note: TT-Tiantai, FX-Fengxian and YX-Yixing.

On Sept. 10, the Yangtze River Delta was affected gradually by the Khanun after its center passed the location of 24.7°N, 124.6°E on the East China Sea at 1800 h. The Yangtze Delta region was within the northwest of the typhoon periphery, where the inverted trough produced the convergent airflow and wind shear (Figs. 1 and 2A). At dusk the weather was fine and the wind speed at 10 m above ground was less than 4 m/s (Fig. 3A and 5A). Under such conditions, the macropterous adult of *N. lugens* was able to emigrate.

**Figure 6:**
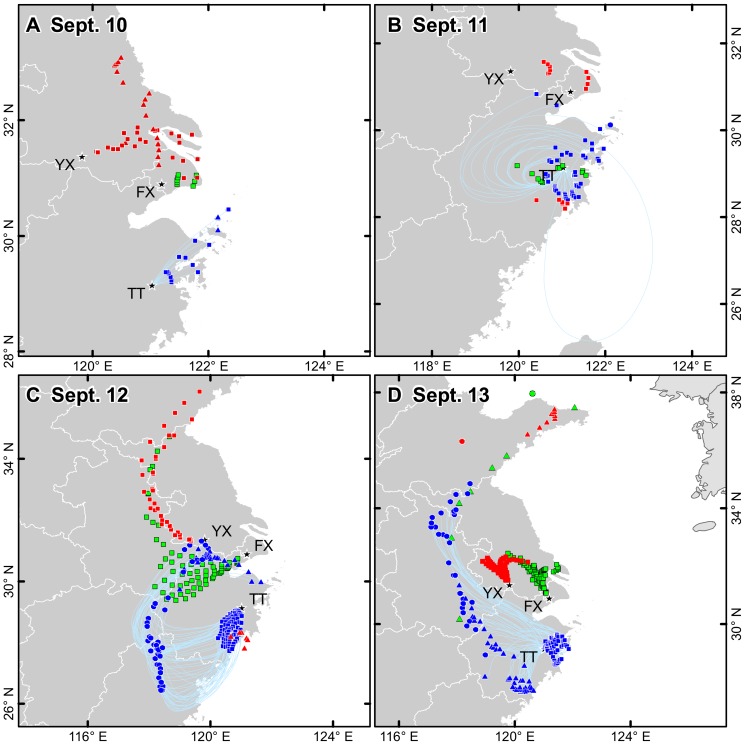
Endpoints of backward trajectories starting from Tiantai (TT), Fengxian (FX) and Yixing (YX) during Sept. 10–13. Note: (i) The endpoints with blue fill color started from Tiantai, those with green fill color started from Fengxian and those with red fill color started from Yixing. (ii)The endpoints in square (▪): the migration duration was less than 13 h, and takes off at 1800 h on the same day; in triangle (▴): the migration duration was 13–24 h, and takes off at 1800 h on the previous day. (•): the migration duration was above 24 h, and takes off at 1800 h on the previous day.

Most light traps in this region experienced dramatic drop on *N. lugens* capture on the night against the increase trend of the past week (Fig. 4). An exceptional catch occurred in Tiantai where the *N. lugens* capture of 26240 individuals is nearly 10 folds of that (2776) on the previous night. There was a lack of distant upwind sources from the analysis of backward trajectories which began from the region (Tiantai, Fengxian and Yixing) and mostly ended in the sea after 3 h flight duration (Fig. 6A). This might explain why most light traps caught less and indicate that the majority adults of *N. lugens* from this region did take-off and fly away. However, the huge captures in Tiantai suggest the place be under a different situation. These captures must have been the airborne population from local or nearby region forced down by the typhoon rain zone approached. On the other hand, the other light traps were further away from the approaching typhoon center and thus the airborne population could have flown away from the region.

### In the vicinity of the Khanun

The Khanun was on the land of eastern China for nearly 30 h from 1450 h on Sept. 11 to 2000 h on Sept. 12. The Khanun made landfall over Luqiao District, Taizhou City, Zhejiang, moved to the northwest, entered Jiangsu at 0415 h in the next early morning, continued in the same direction until the mid-day and then turned towards to the northeast and went to the sea in the evening. The typhoon center had passed near Tiantai, Xiaoshan and Yixing at about 1900, 0000 and 0500 h, respectively. Since the afternoon of Sept. 11, the influence range of the force 10 wind (>24.5 m/s) extended gradually over all the seven PPS except Songyang and Fengxian (Fig. 1).

The Khanun's landfall induced torrential rain in Zhejiang, Shanghai, southern Jiangsu and southeastern Anhui from Sept. 10 to 11. In Zhejiang, about 158 observation stations obtained precipitation of above 100 mm, 61 stations recorded precipitation of above 200 mm, and 10 stations recorded precipitation of above 300 mm in 48 h between 0800 h Sept. 10 and 0800 h Sept. 12. Meanwhile, wind had strengthened in eastern Zhejiang, Shanghai, Jiangsu, and southeastern Anhui since the morning of Sept. 11. Maximum wind speeds ranged from 17.2 m/s to 32.6 m/s, even over 32.6 m/s in some places (Figs. 2B, 2C, 3B, and 5B).

The weather was not suitable for *N. lugens* to take off from this study region at the dusk of Sept. 11, when the typhoon center was near Tiantai and other 6 PPS were located in the gale zone of the typhoon. All the 7 PPS except Tiantai suffered strong winds and heavy rains. Average wind speeds at 10 m above ground ranged from 9 to 11 m/s. However, the simulated hourly precipitation ranged only from 0.2 to 6.1 mm, which was much less than the observed values. By contrast, it was calm in Tiantai when the typhoon eye zone was located nearby at dusk (Figs. 1, 3B, and 5B). Nevertheless, the eye zone was very small, with a diameter only a few kilometres, and it moved very fast. Thus, there was little chance for *N. lugens* to take off in this area, and therefore this region cannot be the source of migrants if there were on the night. All these 7 light traps caught only a few or null *N. lugens* demonstrated that the poor weather conditions prohibited this micro insect's active flight (Fig. 4).

Given that a mass of *N. lugens* airborne was forced to land on the night by the rain, the back trajectories calculated accordingly were nearly all ended in the sea from Tiantai, Fengxian and Yixing. This suggested that if there were some immigrants, they could only have come from nearby areas (Fig. 6B). However, as strong winds and heavy rains must have stopped *N. lugens* from taking off at dusk in nearby regions (Figs. 3B and 5B), therefore, there must have no migrants of *N. lugens* landed in this study region on the night of Sept. 11.

### In the wake of the Khanun

On the next day (Sept. 12) after the Khanun made landfall, its center passed the study region in the early morning (Fig. 1). In the reset of the day and the following day (Sept. 13), the study area was in the rear of the typhoon (Fig. 2D, E, F), where convergence was predominated.

#### On Sept. 12

At dusk, the southern part of the study region was in favourable conditions for *N. lugens* to take off. Fair weather came after the rain, and the winds weakened after the Khanun had passed. It was calm in Zhejiang and Shanghai (Figs. 3C and 5C). However, the average wind speeds at 10 m above ground in Jiangsu was still greater than 4 m/s and thus *N. lugens* take-off was probably suppressed.

The analysis of backward trajectory revealed that the south of the study region might have been the destination other than the source of *N. lugens* migration. Light-trap catches increased sharply in Songyang, Tiantai, Xiaoshan, Zhuji, Fengxian and Yixing on the night (Fig. 4). The endpoints of backward trajectories which originated from Tiantai with flight durations less than 13 h were located in its south-southwest of mostly less than 100 km area. The endpoints with flight durations over 12 h were mostly located at the borders between Zhejiang and neighbouring provinces (Jiangxi, Anhui, Jiangsu and Fujian) (Fig. 6C). However, most of Zhejiang, southern Anhui, southern Jiangsu, northern Jiangxi, and northern Fujian suffered strong winds and heavy rain at dusk on Sept. 11 (Figs. 3B and 5B). Thus these areas must be eliminated from the likely sources. Therefore, catches in Tiantai must have come from short distances where *N. lugens* took off at dusk on the same night. In Fengxian, the endpoints of backward trajectories with flight duration less than 13 h were located in the southwest at the border of Anhui and Zhejiang, where mass take-off of planthoppers was reported on the night as mid-season rice matured and was harvested [13], [16]; while the other longer trajectories were ended in the sea (Fig. 6C). Likewise, the endpoints of backward trajectories from Yixing indicated that the catches of *N. lugens* might have come from as far as up to the northeastern Anhui, northern Jiangsu and southern Shandong (Fig. 6C). However these areas was still under very strong winds at 1800 h, and therefore there should not have too many *N. lugens* able to take off (Fig. 3C). Thus, the catches in Yixing must have come from the surrounding area as well with flight durations less than 12 h (the flight distance in a straight line less than 200 km) (Fig. 6C).

**Figure 7:**
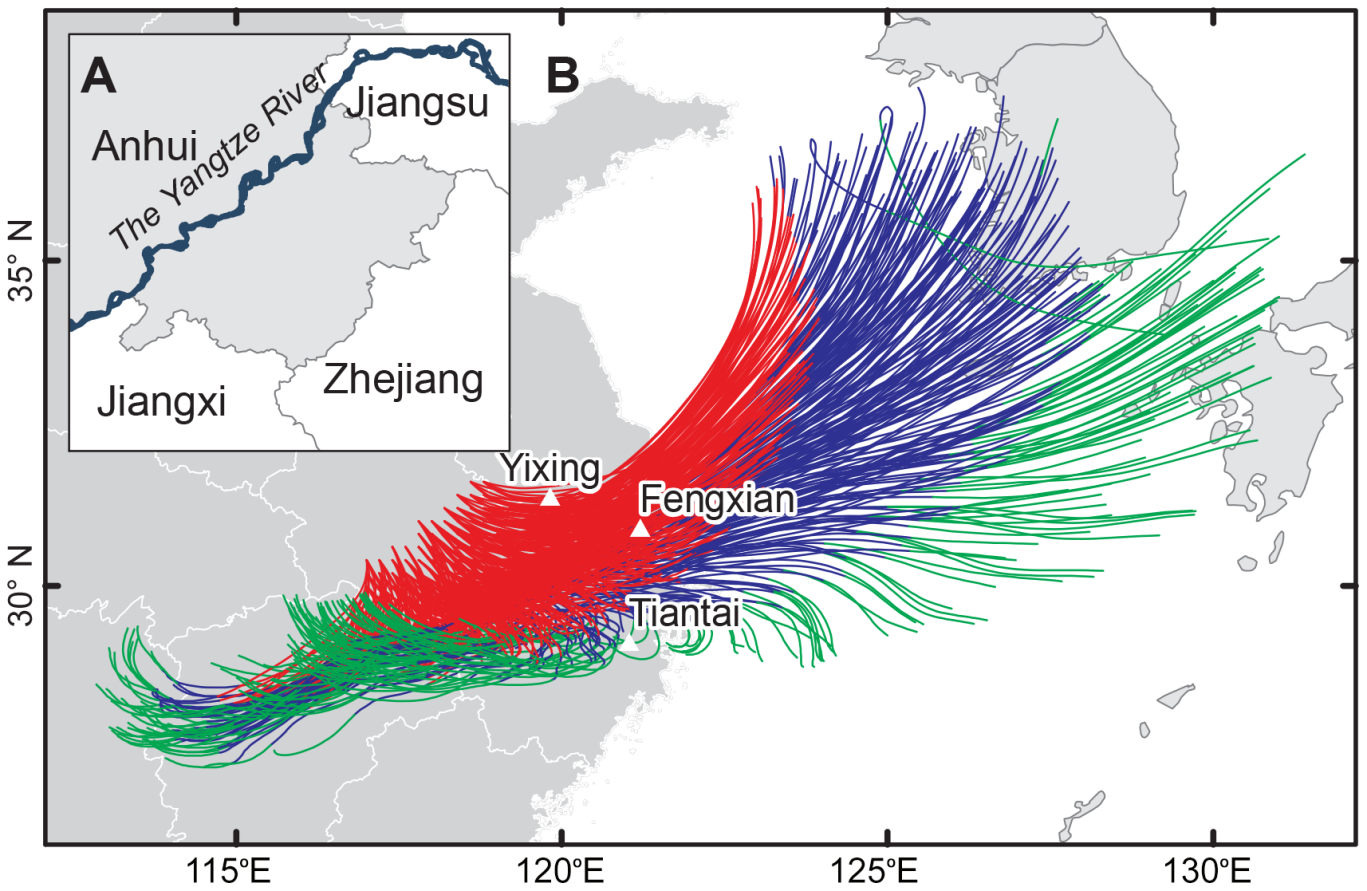
Forward trajectories starting from Southern Anhui at 1800 h on Sept. 12. Note: (i) Start points (+) of forward trajectories from Southern Anhui presented in Fig. (A); (ii) The red lines showed trajectories with flight duration below 12 h, viz. the range that migrants could reach if they flight within one night; (iii) The blue lines showed trajectories with flight duration of 13–24 h, viz. the range that migrants could reach in the daytime of the next day; (iv) The green lines showed trajectories with flight duration of 25–36 h, viz. the range that migrants could reach on the next night.

The analysis of forward trajectory supported the above estimation. In the southern Anhui, it was calm and no rainfall at the dusk on the day (Figs. 3C and 5C). The forward trajectories from the this area suggested that *N. lugens* could have arrived in the southern Jiangsu, northern Zhejiang and Shanghai within 12 h flight (Fig. 7). This agreed very well with the estimates of backward trajectory and indicated that the southern Anhui could have been the source area for immigrants in Yixing and Fengxian on the night.

#### On Sept. 13

The typhoon lost impact on the study region gradually on the day. It had changed to an extratropical cyclone in the Yellow Sea since 0800 h on Sept. 13, and disappeared after 2000 h (Fig. 1). The study region was still in the rear of the typhoon at daytime (Fig. 2E), but under the downdrafts of a continental high pressure which moved slowly towards the southeast due to an anticyclonic shear at nighttime (Fig. 2F).

In contrast to the previous day, the northern part of the study region might be favourable for *N. lugens* to take off at dusk. It was the clear weather with no rainfall and less than 4 m/s wind at 10 m above the ground except the southeastern part of the study region (Tiantai) where was raining lightly at the dusk (Figs. 3D and 5D)

The large numbers of light-trap capture of *N. lugens* indicated more immigrants on the night (Fig. 4). The endpoints of backward trajectories which originated from Tiantai with a flight duration less than 13 h were located in its surrounding area, whereas the endpoints with a flight duration over 12 h were located in Anhui, western and southern Zhejiang (Fig. 6D). Among these regions, the northern Anhui could be excluded as it suffered strong winds at 1800 h on Sept. 12 (Fig. 3C). Therefore, the likely source of the catches in Tiantai was Zhejiang and southern Anhui. The forward trajectories also confirmed that *N. lugens* from the southern Anhui could have got to Tiantai in the daytime on Sept. 13 after more than 12 h flight (Fig. 7). In Yixing and Fengxian, the endpoints of backward trajectories with flight duration under 13 h were located from the northwest in the southern Jiangsu, whereas the endpoints over 12 h were located in Shandong and the sea (Fig. 6D). When the Khanun was on the sea again the Shandong Peninsula suffered rough weather at dusk on Sept. 12 (Figs. 3C and 5C), and few *N. lugens* were able to take off. Therefore, catches in Yixing and Fengxian might have come from the southern Jiangsu, a nearby area (straight-line distance of straight line no more than 200 km), where *N. lugens* might have taken off at dusk on Sept. 13.

From the above study, it was evident that there was much more influx than efflux of *N. lugens* migrants in the Yangtze Delta region during the typhoon Khanun's last 4-day life of Sept. 10–13 2005. Apart from the small amount of adults might have emigrated on the night of Sept. 10 and possibly no migration activity occurred on the next night, a much large amount of *N. lugens* adults must have immigrated on the nights of Sept. 12 and 13. In addition to the residual population, the unusual immigrants during the typhoon Khanun could be the driving force for later outbreak in this region in 2005.

## Discussion

In this study, the return migration of *N. lugens* under the influence of the Khanun was reconstructed using numerical simulations. MM5 has been successfully used to simulate meteorological background in insect migration studies [14], [28], [48], [49]. Although typhoons are difficult to forecast due to their complicated structures and variable routes, MM5 can be used to simulate typhoon processes well based on reanalysis data [50]–[52]. In this study, the simulated trajectory of the typhoon was much better than the previous one obtained by Kou et al. (2009) due to LITTLE_R and FDDA applied with the routine sounding and surface observational data [52]. In spite of this, the results of precipitation intensity prediction by MM5 have not been very satisfactory [51], [52]. It was observed this limitation in this study as well—the precipitation obtained from the numerical simulations was much less than the actual measures [43]. However this would not invalidate the results obtained from this study as wind and temperature are two main factors affecting the transportation of migrating population while rain affects take-off and landing.

Insect migration means persistent and straightened out movement with temporary inhibition of station-keeping response [4], [53]. Generally, *N. lugens* ascend actively straight to sky after take-off, and their phototaxis is inhibited [21], [54]–[56]. Thus, the emigrating planthoppers will not be caught by the local light-traps. During emigration periods, the catches by local light traps are always small except if the emigration is suppressed by bad weather, such as rainfall and downdrafts [13]. This phenomenon has been observed by authors in Yongfu, Guangxi Autonomous Region in last five years (unpublished data) and was evident from this study region on the night of Sept. 10. However, the inhibition of phototaxis lasts only a short time during the ascent process, and the emigrating planthoppers were observed to be caught by local light traps at 33 m above ground [54], [56]. Therefore, planthoppers could be attacted by nearby light traps if they pass the ascent stage.

This study revealed the cause of *N. lugens* later outbreak in the Yangtze Delta region in 2005. Considering the long-distance migration of *N. lugens* often is over 300 km, even 1000 km, the immigrants of *N. lugens* in the Yangtze River Delta during the typhoon Khanun were mainly located in the “nearby” regions of each station (i.e. mostly within 100 km, with a few exceeding 200 km but all less than 300 km) [13], [23], [57]. In other words, the migration of *N. lugens* from the Yangtze River Delta was confined in this area itself by the typhoon Khanun. Compared with their usual emigration destinations (Hunan, Jiangxi and Southern Anhui) in this time of year, *N. lugens* population in the Yangtze River Delta itself did not emigrate successfully. The analyses of trajectory also suggested that the immigrants from the southern Anhui landed in the study region (such as Zhuji, Xiaoshan, Tiantai, Yixing and Fengxian) after the Khanun passed and the resulting population increased (Table 2). This result was agreed with the previous study of Yao et al. (2007), which showed that: (i) the typhoon Khanun resulted in numerous *N. lugens* from the Jiang-Huai Valley landing in Hangjiahu area (located in North Zhejiang, and including Hangzhou, Jiaxing and Huzhou) after the typhoon had passed; and (ii) these immigrants caused the population to increased suddenly [58]. However, the source area of catches in this study was more definitive and more to the southern than in the previous study due to the backward trajectory analysis based on the more accurate meteorological background. This also indicated that typhoons on the East China Sea may not always favour the southward migration of *N. lugens* [35]. The uncommon later immigration of *N. lugens* generated much higher field population in this region.

Insect migration could be terminated by atmospheric environment (such as downdrafts, rain and cold temperature) and other factors (such as topography) [3], [22], [24], [25], [29]–[31]. There has hitherto been only a few examples of landing process observed by radar. In this study, convergence was seemingly the most important factor causing *N. lugens* to be concentrated and deposited. Radar observations have shown that concentrations of migrants form at convergences [2], [4], with densities often increasing as much as 10 to 100 folds. Moreover, this interpretation was also suggested in the previous studies on insect migration influenced by typhoon [58], [59]. In the present study, the failure of *N. lugens* emigration in the Yangtze River Delta was also due to convergence, and under its influence, the emigrants were concentrated and deposited after a short flight duration. In addition, rainfall, strong wind and downdrafts also could suppress insect emigration [13]. In the present case, wind stopped *N. lugens* to take off at dusk on Sept. 12 in Yingxing, while rainfall and downdrafts stopped *N. lugens* to take off at dusk on Sept. 13 in Tiantai.

Overall, this study showed that the return migration of *N. lugens* in the Yangtze River Delta was disrupted and unsuccessful. However, the insects which failed to emigrate could still survive in this region. Nevertheless, the denser population of *N. lugens* would have resulted in faster deterioration of the nutritional status of rice plants and reduction in the survival of the insects. The offspring of the residual and immigrated population would have grown slowly due to deteriorated habitats and declining temperature [60], [61]. Given their slow development, the offspring may have even failed to reach the adult stages and emigrate before rice plants were harvested in mid and late October. Eventually, the offspring of *N. lugens* that failed to emigrate in mid September could not survive and integrate into the overwintering population. Nonetheless, the typhoon Khanun might have caused more damage to crops than its direct impact in this region. After all, this study revealed that the spatial redistribution of *N. lugens* was changed under the influence of the typhoon, leading to populations remaining high in the Yangtze River Delta, and exacerbating outbreaks of this pest, and even affecting the population itself in next year. Compared with the previous work, this study provided more details and improved the understanding on insect migration influenced by typhoon [58], [59].
